# The Current Sphingosine 1 Phosphate Receptor Modulators in the Management of Ulcerative Colitis

**DOI:** 10.3390/jcm14103475

**Published:** 2025-05-15

**Authors:** Xin Yi Choon, Jie Han Yeo, Christopher White, Esha Sharma, Mark A. Samaan

**Affiliations:** 1 Gastroenterology Department, Guy’s and St Thomas’ NHS Foundation Trust, London SE1 7EH, UK; xinyi.choon@gstt.nhs.uk (X.Y.C.); christopher.white5@nhs.net (C.W.); esha.sharma@gstt.nhs.uk (E.S.); 2 Gastroenterology Department, University College London Hospitals, London NW1 2PG, UK; jiehan.yeo@nhs.net

**Keywords:** Sphingosine 1 phosphate receptor modulators, ulcerative colitis, inflammatory bowel disease, ozanimod, etrasimod

## Abstract

Sphingosine 1 phosphate receptor (S1PR) modulators are the latest drug class to have received approval for the treatment of ulcerative colitis, and have brought a new mechanism of action to this landscape. They target immune cell trafficking, specifically the egress of lymphocytes from lymph nodes to the bloodstream, and have proven to be an efficacious and safe anti-inflammatory mechanism. This narrative review aims to distil the key trial data on the efficacy and safety of ozanimod and etrasimod, the two S1PR modulators currently licensed for use in UC. We discuss the higher response rates in the advanced therapy naive versus exposed subgroups. We summarise their safety profiles, taking into consideration open label extension data. Finally, we consider where this class of drugs may be best placed in the treatment landscape and also provide a practical guide for their use in clinical practice.

## 1. Introduction

Ulcerative colitis (UC) is an idiopathic, chronic immune mediated disease that results in colonic inflammation. It has a relapsing–remitting course and has a negative impact on the quality of life (QoL) of patients. Inflammation in IBD is driven by a complex interplay between host genetics, microbiome dysbiosis, a defective epithelial barrier and dysregulated immune responses [1]. Current medical treatments aim to reduce inflammation by targeting circulating lymphocytes, cytokines or the signalling pathways that drive the inflammatory cascade [1].

Current treatments for UC can be broadly classified into conventional therapies (aminosalicylates, immunomodulators and corticosteroids) and advanced therapies (biologics and small molecules). In recent years, there has been a surge in the development of novel therapeutic agents for UC.

Sphingosine 1 phosphate receptor (S1PR) modulators were approved for use in multiple sclerosis (MS) over a decade ago, but have only recently been approved for use in UC [2,3]. Etrasimod is the most recent S1PR modulator to have received regulatory approval, with others in this class undergoing phase 2 and 3 trials [4,5]. With a growing armamentarium of pharmacological options available, it is important that gastroenterologists managing patients with IBD understand the key pivotal trial outcomes for efficacy and safety when using this drug class. Furthermore, as many patients prefer oral therapy over intravenous or subcutaneous formulations, the use of S1PR modulators is likely to grow and familiarity with this class of medications is essential [6].

This review provides a comprehensive overview of these modulators’ mechanisms of action and the pivotal trials demonstrating their efficacy and safety. We also aim to provide a practical guide to the use of S1PR modulators in UC.

### Literature Search

A literature search was conducted in December 2024. The PubMed database was searched using terms related to disease areas: S1P pathway, S1PR modulators, ozanimod, etrasimod, biologic/advanced therapy exposed, biologic/advanced therapy naïve, loss of response and treatment failure. Only articles published in English and from January 2010 were included. Additional key references were identified by searching the bibliographies of retrieved articles.

## 2. Mechanism of Action

### 2.1. S1P Pathway

S1P is a membrane-derived bioactive lipid signalling molecule. It is synthesised intracellularly via sphingosine phosphorylation by sphingosine kinase 1 and 2 (SPhK1, SPhK2) [7]. S1P exerts most of its biological functions by activating five cell-surface G protein-coupled receptors: S1PR 1 to 5 [7]. These five receptor subtypes have roles in the immune, vascular and nervous systems, including the regulation of lymphocyte migration and vascular barrier integrity.

S1PR1 is expressed on lymphocytes and endothelial cells and is the most ubiquitous and well-studied of the five receptors [7,8]. The S1P/S1PR1 interaction mediates the traffic of dendritic, B and T cells—specifically naïve and central memory CCR7-positive T cells, but not the effector memory CCR7-positive T cells involved in tissue immune surveillance [7,8]. The direction of the traffic is determined by the S1P gradient between tissues and the circulatory system [7,8].

**Figure 1:**
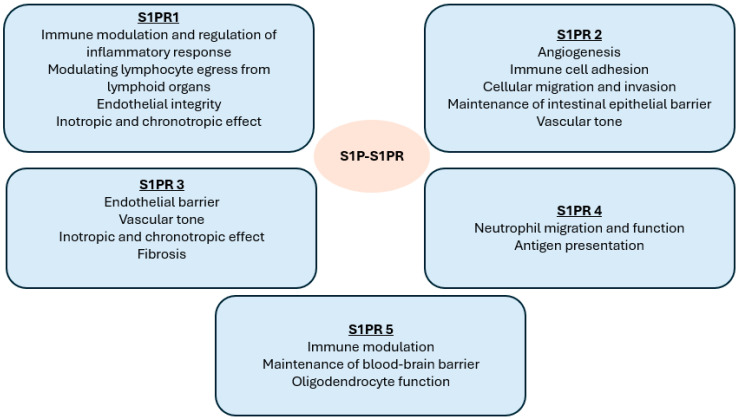
Physiological functions of S1P binding to S1PR subtypes.

The functions of S1PR2 are diverse and its activation has been shown to play a role in inflammatory diseases such as MS and asthma, while its antagonism can lead to the proliferation of diffuse large B-cell lymphoma [9]. S1PR3 has a role in the maintenance of the endothelial barrier and in the mediation of vasoconstriction/vasorelaxation, and, as it is expressed on cardiomyocytes, it has a negative inotropic and chronotropic effect [10]. S1PR 4 is widely expression in lymphoid organs and mediates the activation of dendritic cells in the central nervous system. S1PR4 also has a role in neutrophil function/migration and antigen presentation. S1PR5 mediates natural killer cell trafficking and contributes to the maintenance of the blood–brain barrier [7,8]. Figure 1 summarises the effect of S1P binding to its receptor subtypes.

### 2.2. S1P/S1PR Axis in IBD

The S1P/S1PR axis as a regulator of lymphocyte trafficking plays an important role in the pathogenesis of IBD. S1P binding to S1PR1 promotes lymphocyte migration to intestinal tissue, leading to the infiltration and accumulation of lymphocytes in the gut [7,8]. Beyond lymphocyte egress, S1Ps also play a part in inducing macrophage polarisation, inhibiting the apoptosis of neutrophils and promoting dendritic cell activation, further aggravating the inflammatory process [7,8]. S1Ps are expressed at greater levels at sites of inflammation, due to a dysregulation in the enzymes controlling tissue S1P levels, favouring synthesis over degradation [7,8]. Chronic inflammation also modulates S1PR1 expression [7,8].

There are also studies on the interactions between S1P signalling and both the gut microbiota and intestinal epithelial barrier [11]. Whilst data for the former is limited, studies suggest that upregulation of S1P/SPhK can exacerbate colitis by altering the composition of gut microbiota. However, the role that S1Ps also play in maintaining the epithelial barrier underscores their complicated regulatory function in the intestinal microenvironment.

### 2.3. S1PR Modulation

S1P signalling has thus become a therapeutic target of great interest for immune-mediated conditions over the last decade [7,8]. S1PR modulators, when bound to S1PR, lead to receptor internalisation and degradation, which results in an attenuation of S1P signalling [12,13]. Lymphocytes are thus rendered incapable of following the S1P gradient and are sequestered in the lymph nodes. S1PR modulation has the effect of blocking lymphocyte trafficking to sites of inflammation without affecting global immune function [14]. The first S1PR modulator developed was fingolimod, for relapsing–remitting multiple sclerosis, a structural analogue of S1P with a non-selective pan-S1PR antagonist action [15]. Fingolimod’s ability to ameliorate chronic colitis in preclinical studies of mice models showed promise for S1PR modulation as a novel treatment target in IBD [16]. However, its non-discriminatory effect on all S1PRs is associated with a less favourable cardiac safety profile, with bradycardia occurring in 0.6% of patients in a large real-world study [17]. Off-target effects on S1PR receptor subtypes also led to a greater incidence of other adverse events such as macular oedema (2%) and neoplasms (2%) [18,19]. On this basis, clinical trials in IBD have favoured the more selective S1PR modulators discussed below.

## 3. Ozanimod

Ozanimod was the first in this class to be approved for the treatment of moderate to severe UC [2]. It is a potent S1PR modulator that binds with high affinity to the S1PR 1 and 5 receptor subtypes [20,21]. It has a once-daily formulation, with a median time to maximum drug concentration (Tmax) of 8–12 h and a half-life of 15.8–21 h [21,22,23]. Several enzyme systems are involved in the metabolism of ozanimod, including cytochrome P450, 3A4 and 2C8 and monoamine oxidase B (MAO-B).

### 3.1. Pivotal Trials

TOUCHSTONE was a phase 2 randomised, double-blind, placebo-controlled trial investigating the efficacy of ozanimod against placebo [24]. The study included patients aged 18 to 75 years with moderate to severe UC (Mayo score of 6–12 and an endoscopic subscore of 2 or 3). Whilst stable doses of oral aminosalicylates or prednisone (≤30 mg/day) were permitted, biologics, thiopurines and methotrexate were discontinued for five half-lives prior to enrolment and 4 weeks before screening endoscopy. The primary outcome was clinical remission (Mayo Score ≤ 2 with no subscore > 1 at Week 8).

A total of 197 adult participants were randomised in a 1:1:1 ratio to receive ozanimod 0.5 mg or 1 mg or a placebo; 186 (94%) completed the 8-week induction period. Clinical remission was achieved in 14%, 16% and 6% in the three arms, respectively, with statistical significance observed with ozanimod 1 mg versus placebo (*p* = 0.048). A significantly greater number of patients receiving ozanimod achieved clinical response and mucosal healing compared to the placebo: 57% vs. 37% (*p* = 0.02), 34% vs. 12% (*p* = 0.002) [24]. There was a numerically greater number of patients receiving ozanimod achieving histological remission, but this result was not statistically significant (22% vs. 11%, *p* = 0.07) [24].

The positive signal for efficacy in TOUCHSTONE led to its phase 3 study, True North. This was a randomised, double-blind, placebo-controlled trial that evaluated the safety and efficacy of ozanimod 0.92 mg once daily vs. placebo in patients with moderately to severely active UC [25]. It included adults 18–75 years old with a total Mayo score of 6 −12 with a Mayo endoscopic subscore ≥ 2 and rectal bleeding and stool frequency scores > 1. Corticosteroid doses were fixed during induction, followed by a standardised tapering regime during the maintenance period. Patients were excluded if they had isolated proctitis, failed to respond to induction therapy with ≥2 approved biologic agents for UC, had a clinically relevant cardiac condition, type 1 or poorly controlled type 2 diabetes or a history of uveitis or macular oedema.

True North comprised two cohorts: Cohort 1 consisted of patients randomised in a 2:1 ratio to receive ozanimod 0.92 mg (*n* = 429) or a placebo (*n* = 216), Cohort 2 (*n* = 367) received open-label ozanimod. A 7-day period of dose escalation of ozanimod was used to minimise the risk of bradycardia: 0.25 mg on Days 1 to 4, 0.5 mg on Days 5–7 and 0.92 mg thereafter. Patients with a clinical response at Week 10 (*n* = 457) were re-randomised in a 1:1 ratio to receive either ozanimod or a placebo for the maintenance phase. Prior anti-tumour necrosis factor exposure occurred at 30% in Cohort 1 and 43% of Cohort 2.

**Figure 2:**
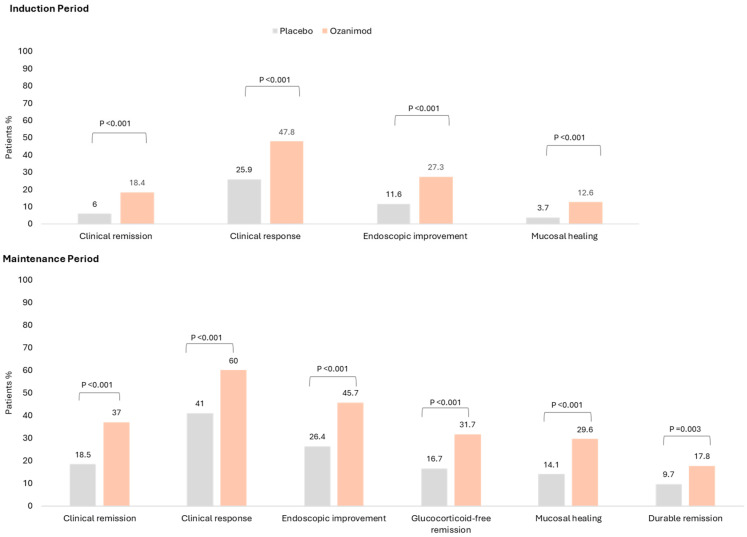
Efficacy results for ozanimod as induction and maintenance, as compared with a placebo (in the ITT population) Reproduced with permission from Sandborn et al., NEJM; Ozanimod as induction and maintenance for ulcerative colitis; 2021 [25].

The double-blind 52-week trial achieved its primary endpoint: Significantly more patients achieved clinical remission with ozanimod vs. placebo for both the 10-week induction phase (18.4% vs. 6.0%; *p* < 0.001) and 42-week maintenance phase (37.0% vs. 18.5%, among patients with a clinical response at Week 10; *p* < 0.001). Furthermore, significantly more patients achieved the secondary endpoints of clinical response (60% vs. 41%; *p* < 0.001), mucosal healing (29.6% vs. 14.1%; *p* < 0.001) and corticosteroid-free remission (31.7% vs. 16.7%; *p* < 0.001) [25]. These results are summarised in Figure 2. A post-hoc analysis demonstrated the rapid onset of action for ozanimod: more patients receiving ozanimod achieved a symptomatic response over the placebo with a delta of 9.6% and 14.0% at Week 2 and 4, respectively [26].

### 3.2. Biologic/JAK Inhibitor Exposed vs. Naïve

Whilst the recent analysis of True North and its open-label extension (OLE) has positioned ozanimod as an effective and well-tolerated treatment for patients naïve to advanced therapies (AT), there remains a paucity of data on AT-exposed patients [27]. True North excluded patients who had failed ≥2 ATs. In a post-hoc analysis, lower rates of Week 10 clinical remission were seen in biologic-exposed patients: clinical remission was achieved in 23% vs. the 6.6% placebo rate in the biologic naïve cohort, 17.2% vs. 8.3% in those with one prior biologic, and 3.7% vs. 2.5% in those exposed to two or more biologics [28]. A similar signal was seen at Week 52, with clinical remission rates of 28% and 26% for patients exposed to one biologic and ≥2 biologics, respectively, compared to 41% in AT-naive patients.

Dignass et al. reviewed both biologic-naïve and exposed clinical non-responders at the end of the induction period and found that whilst there was response in both groups to extended ozanimod induction, higher proportions of biologic-naïve patients achieved a delayed response [29].

Both vedolizumab and S1PR modulators alter lymphocyte trafficking to the gastrointestinal tract, and one may therefore hypothesize that there will be a diminished response to S1P modulation in vedolizumab non-responders. However, a dedicated post-hoc analysis of True North for patients with prior exposure to vedolizumab demonstrated that ozanimod remains an effective treatment option in this group for both inducing and maintaining remission, with clinical remission rates of 39% at Week 52 [28,30]. This maintained efficacy may relate to the disparate mechanisms by which the respective drugs interrupt lymphocyte trafficking.

### 3.3. Longer Term Efficacy and Real-World Data

The phase 2 TOUCHSTONE study was closed in 2019 after all active patients had completed 200 weeks of follow-up [31]. Eligible patients remaining in the study at this point could roll into the phase 3 OLE. Clinical response and remission rates at week 200 were 93.3% and 82.7% for observed cases, and 41.2% and 36.5% when non-responder imputation analysis was employed. For observed cases, histological remission rates were reported at 46.3% and 38.5% at weeks 56 and 104, respectively, with endoscopic improvement rates of 46.4% and 46.5% at these timepoints [31]. Similar results were seen in the True North OLE, with sustained rates of clinical, corticosteroid-free and histological remission rates of 69.3%, 67.9% and 67.3% after 146 total weeks on ozanimod in the Week-52 clinical responders [32]. No new safety signals were identified [32,33], but the risk of rarer adverse events with longer-term use remains to be elucidated.

The current real-world data for ozanimod is limited by the heterogeneity in study designs, follow-up duration and patient populations, and also incomplete data, but still provide valuable information about real-world efficacy and safety signals.

A retrospective study from a multi-centre consortium comprising 146 patients demonstrated the real-world efficacy of ozanimod in inducing clinical remission for both AT-naïve and AT-exposed patients [34]. Week 12 clinical remission rates were 59% in AT-naïve vs. 36% in ≥3 AT-exposed patients. These results are similar to the post-hoc analysis of True North, with lower remission rates in AT-exposed patients.

Cohen et al. performed a prospective real-world study that included 45 patients, with 22 patients having 12-month follow-up data. Ozanimod was effective in inducing and maintaining remission in both AT-naïve and AT-exposed patients [35]. Week 10 clinical remission rates were 57% and 48%, respectively, with Week 52 clinical remission rates of 33% and 26%, respectively.

POLARIS is a phase 4 prospective open-label study aimed at determining the real-world efficacy of ozanimod in both AT-naïve and exposed patients [36]. The study is still ongoing, and an interim analysis demonstrated clinical response and remission rates comparable to the True North studies, with no new safety signals identified.

## 4. Etrasimod

Etrasimod is a once-daily, oral S1PR modulator with selective activation of S1PR1, 4 and 5 [37,38]. It has rapid absorption and its maximum plasma concentration is reached 4 h after ingestion. It has a short half-life of 29.7–36.4 h and is metabolised by three different cytochrome P450 enzymes in the liver.

OASIS was its phase 2 dose-finding study. It was a randomised, double-blind, placebo-controlled study with a primary endpoint of an increase in the mean improvement of modified Mayo Clinical scores (mMCS) at Week 12, which includes stool frequency, rectal bleeding and endoscopic findings. Etrasimod 2 mg met its primary and all its secondary endpoints, with significantly more patients achieving an improved mMCS (∆ = 0.99, *p* = 0.009) and endoscopic improvement (∆ = 24.4%, *p* = 0.003) [39].

The positive results of OASIS paved the way for the pivotal phase 3 studies ELEVATE UC 12 and ELEVATE UC 52. These were two randomised, multi-centre, double-blind, placebo-controlled trials with a treat-through design [40]. Patients were randomised in a 2:1 ratio to receive etrasimod 2 mg OD or a placebo for a 12-week induction period, followed by a 40-week maintenance period (ELEVATE 52 only).

The inclusion criteria for both studies were a modified Mayo score (MMS) of 4–9 with a centrally read endoscopic subscore of ≥2 and rectal bleeding subscore of ≥1. Previous registration trials for biologics/small molecules excluded patients with proctitis, but this patient population was eligible for both ELEVATE UC 12 and 52. They comprised 8.8% and 6.5% of the study population in the two studies, respectively. Both studies excluded patients who were exposed to at least three biological agents or two biological agents and a Janus kinase (JAK) inhibitor. Corticosteroid taper started from week 12 and followed a standardised tapering regime, and concomitant thiopurine therapy was not permitted. Over 50% of each group had a baseline MES of 3 and 37% of the study population had previous AT exposure.

### 4.1. Efficacy

The primary endpoint of ELEVATE UC 12 was the proportion of patients in clinical remission at Week 12 [40]. The co-primary endpoints in ELEVATE UC 52 were the proportion of patients who achieved clinical remission at Week 12 and Week 52. Etrasimod was able to achieve its primary and secondary endpoints in both ELEVATE studies. The results from the induction and maintenance studies are shown in Table 1 and Table 2, respectively.

In ELEVATE UC 52, endoscopic improvement, symptomatic remission and endoscopic improvement–histological remission were also significantly greater in the etrasimod group over placebo with a delta of 26.7%, 24.9% and 18.4%, respectively. There was a statistically significant improvement in symptom burden in the etrasimod group over the placebo in both ELEVATE UC 12 and 52, with a reduction in rectal bleeding and stool frequency subscores as early as Week 2. Post-hoc analysis of both studies demonstrated the efficacy of etrasimod over the placebo for isolated proctitis, with Week 52 clinical remission in 44% of etrasimod- vs. placebo-treated 11%, *p* < 0.001 [41]. There was also a significant difference for corticosteroid-free clinical remission in the etrasimod group (etrasimod 44% vs. 11.1% placebo, *p* < 0.001). However, a significant difference between etrasimod and the placebo was not seen across all secondary endpoints, which is likely due to the small sample size (*n* = 64 with proctitis in both groups). Although there are limitations to post-hoc analyses, this expands upon the known efficacy of etrasimod in a subpopulation of UC patients who often have a heavy symptom burden and have historically been excluded from clinical trials.

### 4.2. Biologic/JAK Inhibitor-Exposed vs. Naïve

Vermeire et al. performed a post-hoc analysis of the ELEVATE programme to assess the impact of prior AT on the efficacy of etrasimod [42]. Etrasimod remained effective and superior to placebo in achieving clinical remission in both AT-exposed and naïve patients. However, the rates of clinical remission at Week 12 were lower in those with AT exposure (17.5% vs. placebo 2.4%, *p* = 0.004) compared to the naïve cohort (30.9% vs. placebo 9.7%, *p* < 0.001). A similar result was seen at Week 52, with a greater proportion of AT-naïve patients in clinical remission (36.6% vs. placebo 7.5%, *p* < 0.001) compared to the exposed cohort (21.3% vs. placebo 4.8%, *p* = 0.011).

In the same post hoc analysis, the efficacy of etrasimod in vedolizumab-exposed patients was explored. There was a non-significant numerical improvement for etrasimod relative to placebo for the majority of the primary and secondary points [42]. This is in contrast to the results from a post-hoc analysis of True North where ozanimod was superior to the placebo in vedolizumab-exposed patients [28]. As with other post-hoc analyses, this result is limited by a small sample size, with only 11% of patients in the ELEVATE studies having prior exposure to anti-integrin therapy, and therefore should be interpreted with caution.

### 4.3. Real-World Data

At the time of writing this review, there were no published real-world studies. An international, multi-centre, non-interventional study to assess the efficacy and safety of etrasimod is ongoing (NCT06294925), with its primary endpoint being clinical remission at Week 12 and 52. This study will shed more light on the real-world efficacy/safety of etrasimod and is estimated to finish in 2027 [43].

## 5. Safety

An important consideration prior to the initiation of any immunosuppressive therapy is safety. S1PR modulators have shown a favourable safety profile in their phase 2 and 3 trials [38]. The adverse effects of S1PR modulators relate to their interactions with S1PR1 and S1PR5 in various tissues [38]. In True North, adverse events occurred in 37–49% patients in the ozanimod and placebo groups [25]. There was a higher rate of adverse events in the ELEVATE studies, but the rates were comparable between the etrasimod and placebo groups (71% vs. 81%, respectively) [40].

This next section will cover the current safety data available.

### 5.1. Cardiac Adverse Events

S1PR1 is expressed by cardiac tissue, and following receptor activation, an intracellular shift in potassium occurs leading to reduced excitability and transient bradycardia. This occurs in a dose-dependent manner [38]. These effects can be mitigated with gradual dose escalation for ozanimod, but this is not required for etrasimod [25,40,44].

Bradycardia: Symptomatic bradycardia occurred in five (0.6%) patients in the treatment arm of the True North study [25]. Of these, only one was symptomatic and resolved following drug discontinuation without the need for additional intervention. No patients in the True North study developed a second or third-degree atrioventricular (AV) block. Across the ELEVATE UC trials, nine occurrences of bradycardia (<1%) were observed in the treatment arms [40]; two of these were symptomatic and led to study discontinuation. Two cases of first-degree AV block and one case of Mobitz type 1 AV block occurred. All three cases of AV block resolved following treatment discontinuation without the need for additional intervention. Overall, the risk of cardiac conduction disorders with the use of S1PR modulators is low in appropriately selected patients.

Hypertension: Eleven patients (1.5%) receiving etrasimod developed hypertension in the ELEVATE studies, but all of these were mild and did not lead to study discontinuation [40]. Similar results were seen in the True North studies [25]. Hypertensive crisis occurred in one patient receiving ozanimod, but this did not lead to treatment discontinuation.

### 5.2. Ophthalmic Adverse Events

S1PR1 is highly expressed in endothelial cells and plays a vital role in regulating vascular permeability [38,45]. S1PR modulators can disrupt this delicate balance and cause macular oedema. In the trials, macular oedema occurred in three patients receiving ozanimod and two patients receiving etrasimod [25,40]. All bar one discontinued treatment, leading to resolution of the macular oedema (one patient receiving etrasimod continued without treatment interruption).

### 5.3. Infections

Safety data from True North noted a higher rate of infections with ozanimod over a placebo during the maintenance phase, with nasopharyngitis being the most frequent adverse event (3.0% vs. 1.8%) [25]. The rates of serious infections were low, with higher rates seen in the placebo group (1.8% vs. 0.9%). Rates of serious and opportunistic infections were similar between the etrasimod and placebo groups. This reassuring signal is echoed in the respective OLEs [31,32,46].

Herpes Zoster: In True North, the rate of herpes zoster infections was 0.4% and 2.2% in the induction and maintenance phases, respectively, with no events occurring in the placebo group [25]. Four patients in the ELEVATE studies had herpes zoster infections (two receiving etrasimod, two receiving placebo) [40]. None of these adverse events led to discontinuation of the study drug.

Lymphopenia: In keeping with their mechanism of action, a reduction in the absolute lymphocyte count occurred, with a 50% reduction from baseline values by Week 2 and Week 10 in patients receiving etrasimod and ozanimod, respectively [25,40]. The resulting lymphopenia is reversible, with lymphocyte counts returning to baseline within 2–8 weeks of treatment cessation. No patients with an absolute lymphocyte count of less than 0.2 × 10^9^/L developed a serious or opportunistic infection. However, both manufacturers suggest treatment interruption when absolute lymphocyte counts are below 0.2 × 10^9^/L and to consider reinitiation when it is above 0.5 × 10^9^/L [47,48].

### 5.4. Hepatic Adverse Events

Elevated liver aminotransferase levels were more common with ozanimod treatment than with placebo [25]. Abnormal liver function tests led to discontinuation of ozanimod therapy in 0.4% of patients in both the induction and maintenance periods. Similar results were seen in the ELEVATE studies, with two patients (0.5%) in ELEVATE UC 52 discontinuing etrasimod due to elevated alanine aminotransferases [40]. No patients in either registration trials met Hy’s law.

The respective manufacturers recommended performing liver function tests at 1 month after initiation and then 3-monthly for the first year [47,48]. No dose adjustment is required for patients with mild or moderate hepatic impairment, but S1PR modulators should be avoided in patients with severe hepatic impairment (Child–Pugh class C).

### 5.5. Neurological Adverse Events

There has only been one reported case of progressive multifocal leukoencephalopathy (PML) in a patient with MS who was treated with ozanimod [49]. No cases of PML have been reported in the UC population to date.

### 5.6. Malignancies

One case of basal cell carcinoma was diagnosed during the induction phase of True North in a patient receiving ozanimod [25]. During the maintenance period, cancer was diagnosed in four (<0.9%) patients and included two cases of basal cell carcinoma, two cases of colorectal cancer and one of breast cancer. No cases of malignancy were reported in the ELEVATE studies [40].

The manufacturers advise that patients who are initiated on etrasimod or ozanimod should be cautioned against exposure to sunlight without UV protection and that this class of medication should be avoid in patients with active malignancy [47,48].

### 5.7. Pregnancy and Breastfeeding

Etrasimod and ozanimod are both contraindicated in pregnancy, with animal studies demonstrating reproductive toxicity including foetal loss and a higher rate of congenital malformations [50,51,52]. Effective contraception should be used during treatment. If pregnancy is desired, the manufacturers recommend stopping etrasimod at least 14 days and ozanimod 3 months before conception [47,48].

Both ozanimod and etrasimod are excreted in breastmilk in animal studies [50,51,52]. Given the risk for serious adverse reactions in infants, breastfeeding should be avoiding while undergoing treatment.

## 6. Future Perspectives

Several other S1PR modulators are in development for both UC and Crohn’s disease. These range from molecules in the pre-clinical phase to ongoing phase 2 studies. Amiselimod and tamuzimod are examples of S1PR modulators that have been investigated in phase 2 trials [53,54].

Tamuzimod was investigated in a phase 2, multi-centre, randomised, double-blind, placebo-controlled study for patients with moderate to severely active UC [53]. It met its primary endpoint of clinical remission with superiority over placebo (27.9% vs. 11.4%, *p* = 0.0184). There were no new safety signals identified. Long-term and open-label extension phases of the study are ongoing.

Long term extensions and phase 4 studies of ozanimod and etrasimod will provide additional data regarding long term efficacy and safety in ulcerative colitis. In contrast to ulcerative colitis, the phase 3 YELLOWSTONE trial investigating the efficacy of ozanimod in Crohn’s disease has been terminated early due to futility [55]. A phase 2/3 study evaluating the efficacy of etrasimod in Crohn’s disease is ongoing (NCT04173273) [56].

## 7. Treatment Positioning

A systematic review and meta-analysis by Solitano et al. found that S1PR modulators are effective at inducing remission, with low heterogeneity observed between studies [57]. They are also effective at maintaining clinical remission and mucosal healing over placebos. However, there have not been head-to-head trials of S1PR modulators against other agents for ulcerative colitis, and data on treatment positioning is scarce.

When considering where to place S1PR modulators amongst the other treatment options available for UC, the factors that need to be taken into consideration include prior AT exposure, preferred route of administration, comorbidities, extra-intestinal manifestations and local prescribing restrictions [38,58]. The positioning of S1PR modulators in the treatment paradigm is still uncertain, and there is no official guidance on this. The American Gastroenterological Association (AGA) considers this drug class as ‘higher efficacy’ in the AT-naïve cohort, and ‘lower efficacy’ after exposure to one or more advanced therapies [59]. Network meta-analyses (NMA) suggest that both ozanimod and etrasimod remain viable treatment options even in the AT-exposed cohort [60,61]; however, they are by no means a direct comparison of efficacy between therapeutic classes and should be interpreted with caution.

It is our opinion that ozanimod and etrasimod could be positioned either after the first course of oral corticosteroids or after failure/loss of response to conventional therapies (i.e., 5-aminosalicylic acid and/or immunomodulators). They are a once-daily treatment with an oral formulation, which many patients find favourable, and it eliminates the need for infusions, thereby reducing healthcare utilisation [62]. They appear to be more effective in the AT-naïve cohort, and thus may be more suitable as a first-line therapy. There is no risk of immunogenicity nor the need for weight-based dosing. The fast onset of action results in rapid symptom relief and potentially minimises the use of corticosteroids as a bridge to maintenance treatment. The cost of the production of small molecules is significantly less than biologic therapies, which in turn is reflected in the drug acquisition cost, and this will have implications for both publicly and privately funded healthcare systems [63].

Further studies are required to explore the utility of S1PR modulators in patients with extra-intestinal manifestations or concomitant immune mediated conditions. At present, this drug class is approved for use in ulcerative colitis and multiple sclerosis only, but there is emerging evidence of the significance of the S1P/S1PR axis in other autoimmune diseases such as systemic lupus erythematous and Sjogren’s syndrome [64,65].

### A Practical Guide to Using S1PR Modulators

A baseline electrocardiogram (ECG) should be obtained in all patients to assess for pre-existing cardiac abnormalities, and first-dose cardiac monitoring should be performed for certain groups of patients (e.g., history of myocardial infarction). Patients starting ozanimod should undergo an ophthalmic assessment before treatment, and post-initiation assessments are recommended for patients with risk factors for macular oedema (e.g., diabetes mellitus, uveitis). For etrasimod, an ophthalmic assessment prior to initiation is only required for patients with risk factors, and an ophthalmic assessment within 3 months of starting is recommended for patients without these risk factors.

Physicians need to take into account drug–drug interactions and caution is required if patients are taking concomitant cytochrome 450 enzyme inhibitors or inducers or monoamine oxidase B inhibitors. As with all other advanced therapies, live vaccines are contraindicated (including the live varicella zoster vaccine), and the initiation of ozanimod or etrasimod should be delayed for 4 weeks after vaccination [47,48].

**Figure 3:**
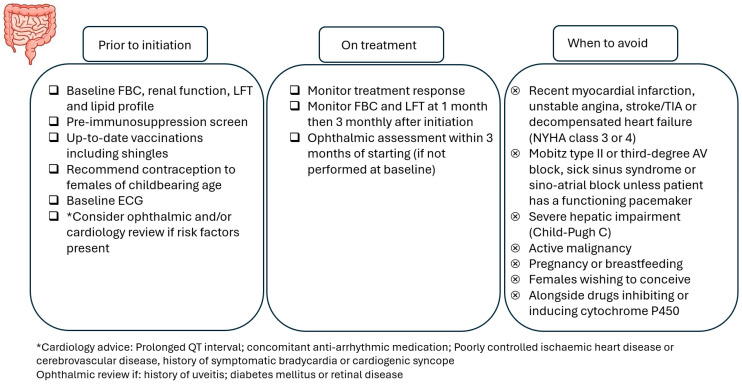
Important considerations for S1PR modulators and contraindications to their use.

Figure 3 highlights the important considerations when using S1PR modulators. 

## 8. Conclusions

S1PR modulators are an emerging therapeutic class in the treatment of UC. They are an oral once-daily therapy with a rapid onset of action. They represent a key line of effective therapy for patients with active disease, with several advantages over monoclonal antibodies. The safety of this class of treatment is promising, especially with appropriate vaccinations and screening tests. More studies will be needed to further understand its efficacy in real-world clinical practice and its positioning in the treatment of UC.

## Figures and Tables

**Table 1:** Primary and secondary endpoints from induction studies. Data from Sandborn et al., Lancet; Etrasimod as induction and maintenance therapy for ulcerative colitis (ELEVATE); 2023 [40]. Clinical remission defined as a composite of stool frequency subscore = 0 (or stool frequency subscore = 1 with a ≥1 point decrease from baseline), rectal bleeding subscore = 0, and endoscopic subscore of ≤1). Symptomatic remission defined as stool frequency subscore = 0 (or stool frequency = 1 with a ≥1 point decrease from baseline). Endoscopic improvement: endoscopic subscore ≤ 1, without friability. Endoscopic improvement–histological remission defined as: endoscopic subscore ≤ 1, without friability) with histological remission (Geboes Index score < 2·0).

	ELEVATE UC 12			ELEVATE UC 52		
	Etrasimod *n* = 222	Placebo *n* = 112	*p* Value	Etrasimod *n* = 274	Placebo *n* = 135	*p* Value
Clinical remission	55 (25%)	17 (15%)	0.026	74 (27%)	10 (7%)	<0.0001
Endoscopic improvement	68 (31%)	21 (19%)	0.009	96 (35%)	19 (14%)	<0.0001
Endoscopic improvement-histologic remission	36 (16%)	10 (9%)	0.036	58 (21%)	6 (4%)	<0.0001
Symptomatic remission	104 (47%)	33 (29%)	0.0013	126 (46%)	29 (21%)	<0.0001

**Table 2:** Primary and key secondary endpoints in the maintenance study. Data from Sandborn et al., Lancet; Etrasimod as induction and maintenance therapy for ulcerative colitis (ELEVATE); 2023 [40].

ELEVATE UC 52			
	Etrasimod *n* = 274	Placebo *n* = 135	*p* Value
Clinical remission	88 (32%)	9 (7%)	<0.0001
Sustained clinical remission	49 (18%)	3 (2%)	<0.0001
Corticosteroid free remission	88 (32%)	9 (7%)	<0.0001
Endoscopic normalization	72 (26%)	8 (6%)	<0.0001

## Data Availability

No new data were created or analysed in this study. Data sharing is not applicable to this article.

## Abbreviations

The following abbreviations are used in this manuscript:

AT	Advanced therapy
FBC	Full blood count
ECG	Electrocardiogram
MDPI	Multidisciplinary Digital Publishing Institute
IBD	Inflammatory bowel disease
ITT	Intention to treat
LFT	Liver function tests
NHS	National health service
OD	Once daily
OLE	Open label extension
S1P	Sphingosine 1 phosphate
S1PR	Sphingosine 1 phosphate receptor
SPhK	Sphingosine kinase
UC	Ulcerative colitis
